# A needle in a haystack: Assessing the radiographic detectability of microsurgical suture needles using a human cadaveric model

**DOI:** 10.1016/j.jpra.2026.01.033

**Published:** 2026-01-30

**Authors:** Lukas P. O’Brien, Kasie O’ Reilly, Mary Ellen McMahon, Antonina Tcacenco, Ronan Cahill, Barry O’Sullivan, Shirley M. Potter

**Affiliations:** a Department of Plastic and Reconstructive Surgery, Mater Misericordiae University Hospital, Dublin, Ireland; b Department of Plastic and Reconstructive Surgery, Beaumont Hospital, Dublin, Ireland; c Department of Plastic and Reconstructive Surgery, St James’s Hospital, Dublin, Ireland; d School of Medicine, University College, Dublin, Ireland

**Keywords:** Cadaver, Microsurgery, Needle, X-ray, Plastic, Surgery

## Abstract

Intraoperative loss of surgical needles represents a rare but significant challenge in operative settings, particularly in microsurgical procedures involving free tissue transfer where fine sutures (8–0 to 10–0) are employed. Current hospital protocols typically use intraoperative portable radiography to localize misplaced needles. However, the diagnostic utility of X-ray imaging in detecting small-caliber needles remains poorly defined.

Following ethical approval, a cadaveric study was conducted using a range of monofilament Ethilon™ needles (3–0 to 10–0 caliber), which were inserted at four anatomically relevant surgical sites. Anteroposterior radiographs were obtained using a standard intraoperative C-arm. A total of 53 participants - including consultant surgeons, radiology trainees, nursing staff, and surgical registrars—were asked to identify needle number and location using blinded image review. Radiographic identification of needles was accurate for calibers 3–0, 4–0, 5–0, and 6–0, with identification rates approaching or exceeding 99%. Smaller needle calibers showed a dramatic decline in detection accuracy. The 8–0 needles were correctly localized in 3.77% of attempts, just one 9–0 needle was successfully identified. None of the 10–0 needles were detected across all anatomical sites.

While portable radiography remains a reliable modality for locating surgical needles of caliber 3–0 to 6–0, it proves ineffective for the detection of finer microsurgical needles (≥8–0). These results have important implications for operative protocols and clinical decision-making. The routine X-ray imaging in cases of microsurgical needle loss warrants reconsideration to favor evidence-informed strategies that minimize intraoperative delays, prolonged anesthesia and unnecessary radiation exposure.

## Introduction

Since the advent of microsurgical techniques, free tissue transfer has become increasingly central to complex reconstructive procedures. These surgeries rely on delicate instruments and ultra-fine suture materials—often ranging from 8–0 to 10–0 in caliber—which present significant challenges when inadvertently lost during surgery. Although rare, retained suture needles can result in postoperative complications and patient anxiety, and their potential medicolegal implications warrant attention.^1^, ^2^, ^3^ Despite this, there is a paucity of robust evidence supporting the effectiveness of intraoperative imaging in locating such fine-caliber needles.^2^, ^3^, ^4^ Typically, these items are only recognized as missing during final surgical counts, complicating efforts to recover them as retained surgical items (RSIs). While the theoretical risks of retained needles are acknowledged, literature reviews indicate no cases necessitating return to theatre solely for retrieval of 9–0 or 10–0 needles due to anticipated complications.^1^^,^^4^

In Ireland, the Health Service Executive (HSE) mandates open disclosure of RSIs, requiring clinicians to inform patients of potential harm, regardless of whether any injury has occurred.^5^ Although not exclusive to microsurgery, RSIs are well-documented across various specialties including ophthalmology, gynecology, vascular and general surgery.^3^^,^^6^^,^^7^ Reported incidence rates range from one in 1000 to one in 19,000 surgeries, with surgical swabs being the most commonly retained items and needles less frequently reported.^7^ A meta-analysis by Moffatt-Bruce et al. identified risk factors such as elevated BMI, emergency operations, blood loss exceeding 500 mL, and surgical count discrepancies as correlates of RSI occurrence.^8^ Retained surgical instruments occur more frequently in emergency procedures and in operations complicated by unexpected intraoperative changes. Potential risks include surgical site infection with potential progression to sepsis, injury to adjacent anatomical structures (such as nerves and blood vessels), chronic inflammation with subsequent fibrosis or scarring, hemorrhage, and persistent postoperative pain.^6^^,^^9^

A clinical survey by McMahon et al. found that 92.5% of reconstructive surgeons had experienced microsurgical needle loss intraoperatively, yet only 15.8% used imaging—and none were successful in localizing the lost needle.^9^ This discrepancy underscores the limitations of current detection methods and highlights the need for further research into effective retrieval strategies.

Imaging modality and needle caliber are crucial in determining visibility. Case reports have demonstrated that small needles (e.g., 8–0) may go undetected intraoperatively on X-ray, but may later be identified using MRI or CT, often prompting reoperation.^10^^,^^11^ This raises a critical question regarding the trade-off between pursuing retrieval using radiographic methods versus accepting the theoretical risks of retention. Particularly, the risk of complications such as pneumothorax or pneumoperitoneum may influence clinical decisions about whether to leave a needle in situ.^12^ Macilquham et al. showed that while 17 mm (5–0) needles were consistently visible, only 13% of participants could detect 13 mm needles.^2^ The current study is based on institutional protocol at the senior author’s center, which mandates intraoperative radiography in cases of needle count discrepancy.

This study aims to evaluate the diagnostic utility of intraoperative radiography in detecting retained microsurgical suture needles. Specifically, it examines needle calibers 3–0 to 10–0, with a focus on the smaller 8–0, 9–0, and 10–0 sizes (6.5 mm, 3.5 mm, and 5.1 mm, respectively).^2^ By utilizing a cadaveric model and a controlled imaging protocol, this study seeks to provide evidence-based guidance on the limitations of radiographic detection and inform surgical protocols for needle loss during microsurgical procedures.

## Methods

Ethical approval for this study was obtained from both the Mater Misericordiae University Hospital (MMUH) and University College Dublin (UCD). A formalin-embalmed cadaver was obtained from the UCD School of Medicine and transported to MMUH, where the study was conducted in a decommissioned surgical theatre to replicate a realistic operative setting. This location allowed the research team to simulate operating theatre conditions while maintaining strict control over environmental variables and imaging protocols.

Four anatomical locations frequently encountered in microsurgical free flap reconstruction were selected: the distal tibia, distal radius, anterior chest (second intercostal space), and anterior neck. These sites were chosen due to their clinical relevance and their differing tissue compositions, which allowed evaluation of imaging performance under varied soft tissue and bony backgrounds.

Two investigators (LOB and SP), both experienced in microsurgery, randomly inserted seven Ethilon™ (Ethicon, Johnson & Johnson) suture needles of varying calibers—3–0 to 10–0—into subcutaneous tissue at each anatomical site (see Table 1). Each needle was placed using forceps at a consistent depth of approximately 5–10 mm beneath the skin surface to mimic real intraoperative loss conditions. Needle orientations varied randomly to reflect the variability encountered during surgery.

**Table 1:** Demonstrating suture needle caliber, arc and length.

Needle	Arc	Length
3–0	3/8	19 mm
4–0	3/8	19 mm
5–0	3/8	19 mm
6–0	3/8	16 mm
7–0	3/8	11 mm
8–0	3/8	6.5 mm
9–0	3/8	6.5 mm
10–0	3/8	6 mm

Following needle placement, anteroposterior (AP) radiographs were obtained using a standard mobile C-arm fluoroscopy unit operated by a licensed radiographer. Imaging parameters were individually optimized for each anatomical site to maximize visibility of small metallic objects while minimizing radiation exposure, in line with standard intraoperative practices:

**Table:** 

Lower leg	54 kVp	3.6 mAs
Forearm	49 kVp	0.5 mAs
Thorax	64 kVp	1.6 mAs
Face-neck	66 kVp	3.0 mAs

Automatic exposure rate control was employed to modulate kilovoltage peak (kVp) and milliampere-seconds (mAs), ensuring a balance between image clarity and dose efficiency. Manual exposure was trialed for one radiograph but yielded no improvement in image quality. Radiographic best practices were followed, including minimizing object-to-detector distance, employing tight collimation to reduce scatter, and ensuring optimal focus-to-skin distance to enhance resolution and reduce geometric blurring.

To facilitate accuracy in evaluating needle detection, larger straight radiopaque needles were subsequently placed at each site and imaged to serve as reference markers for later comparison. These needles were used to delineate the regions of interest where the microsurgical needles were embedded. A total of four images were acquired per anatomical site:

**Table:** 

A	Photograph of the cadaveric anatomical site.
B	Baseline radiograph without any needle placement (control image).
C	Radiograph with all seven microsurgical needles placed.
D	Radiograph with the same seven needles plus reference straight needles.

**Figure 1:**
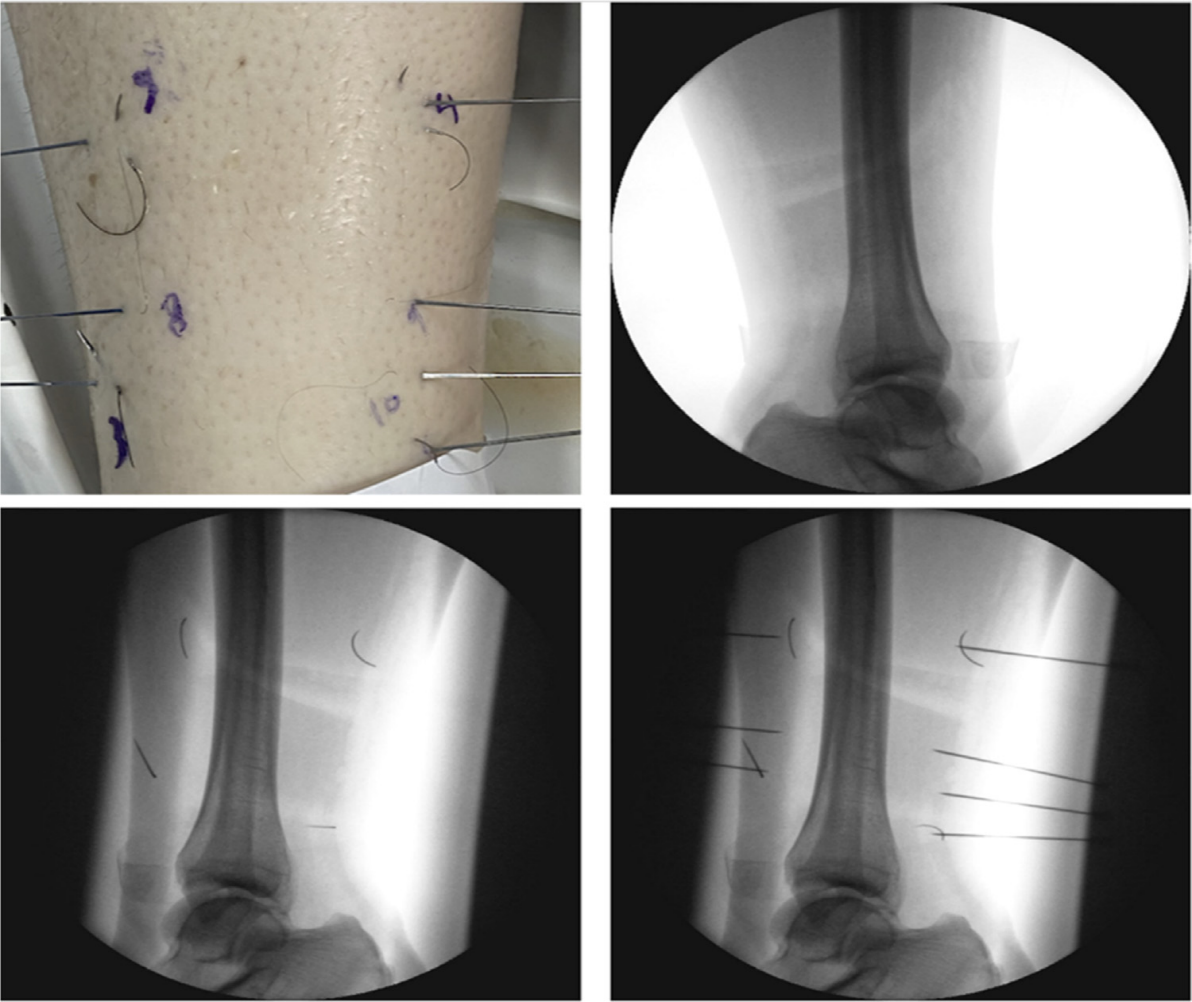
Distal tibia images/radiographs for needle identification.

**Figure 2:**
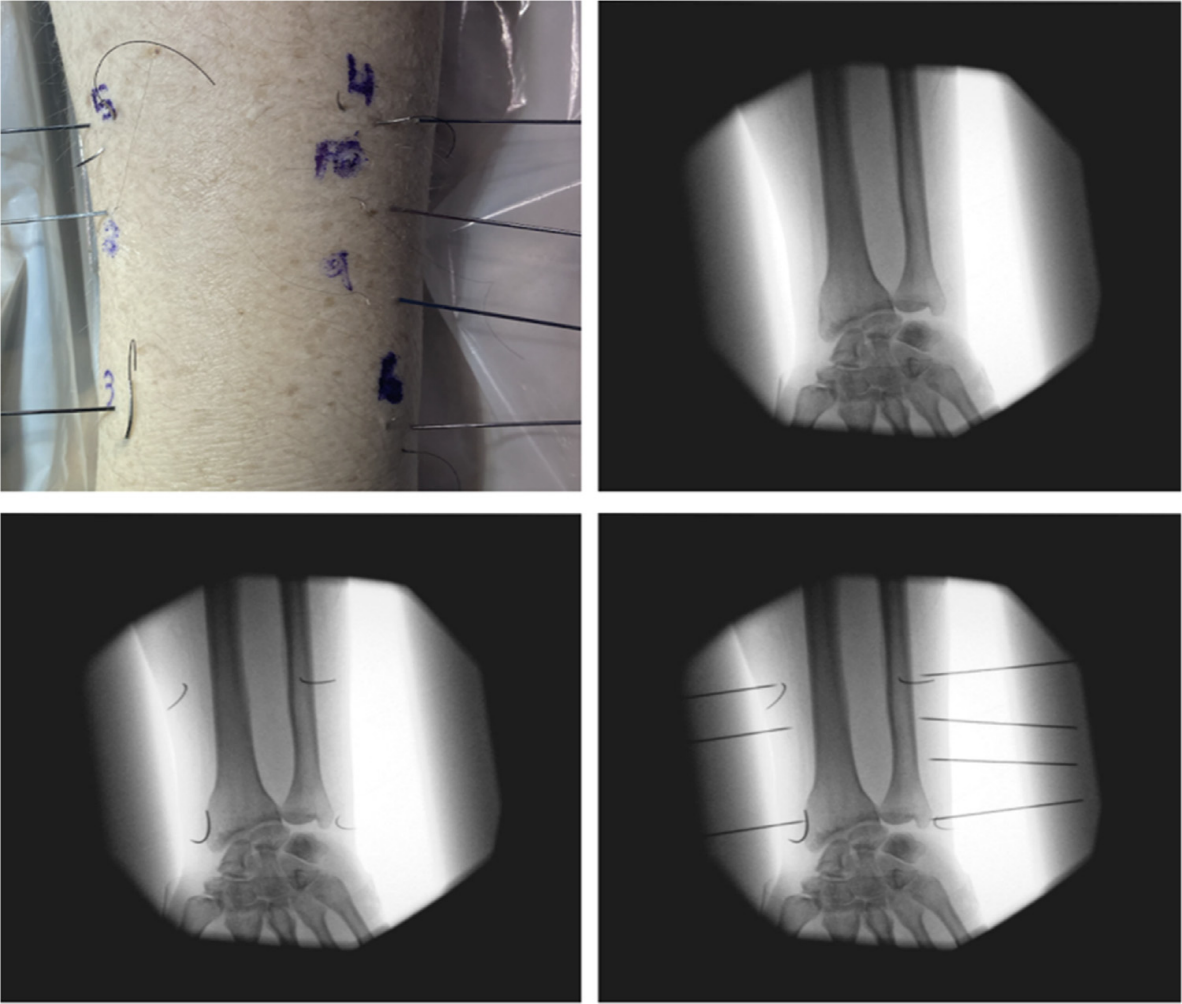
Distal Radius images/radiographs for needle identification.

**Figure 3:**
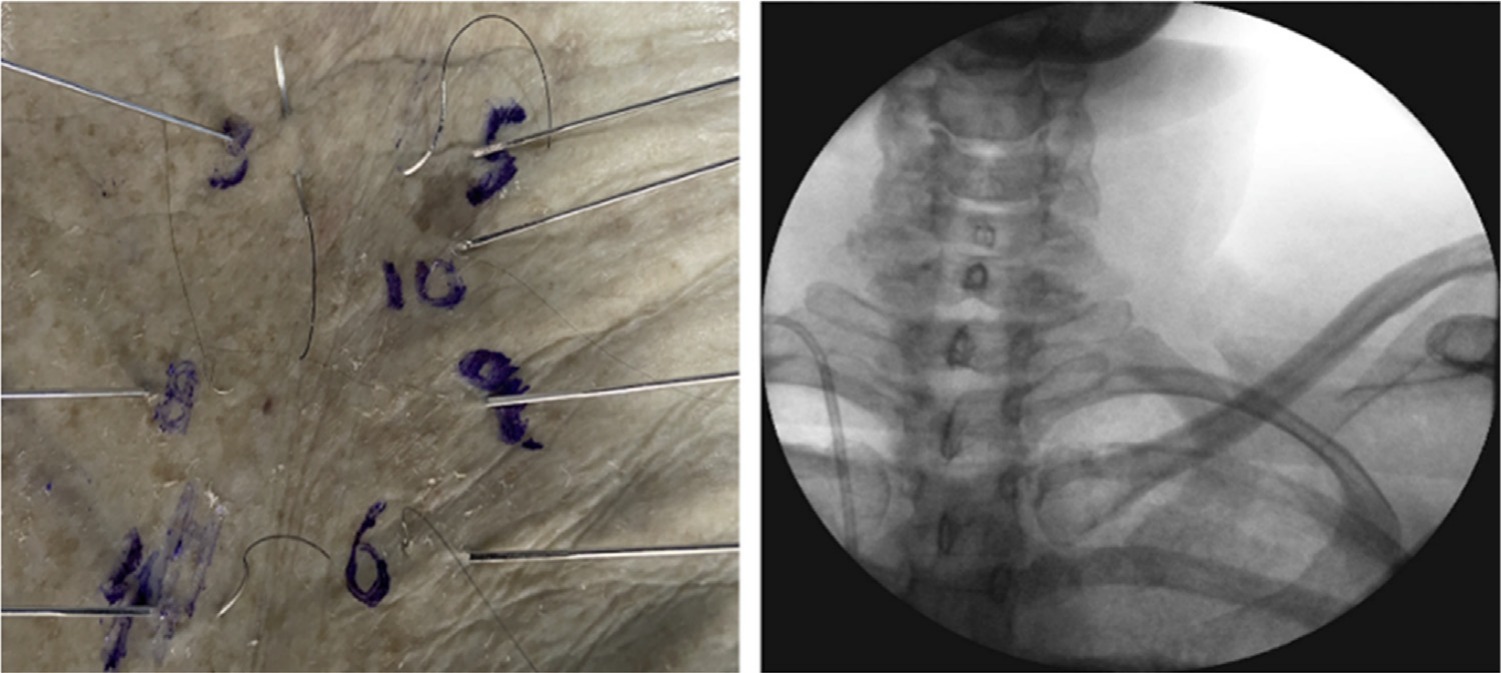
Second intercostal space images/radiograph for needle identification.

**Figure 4:**
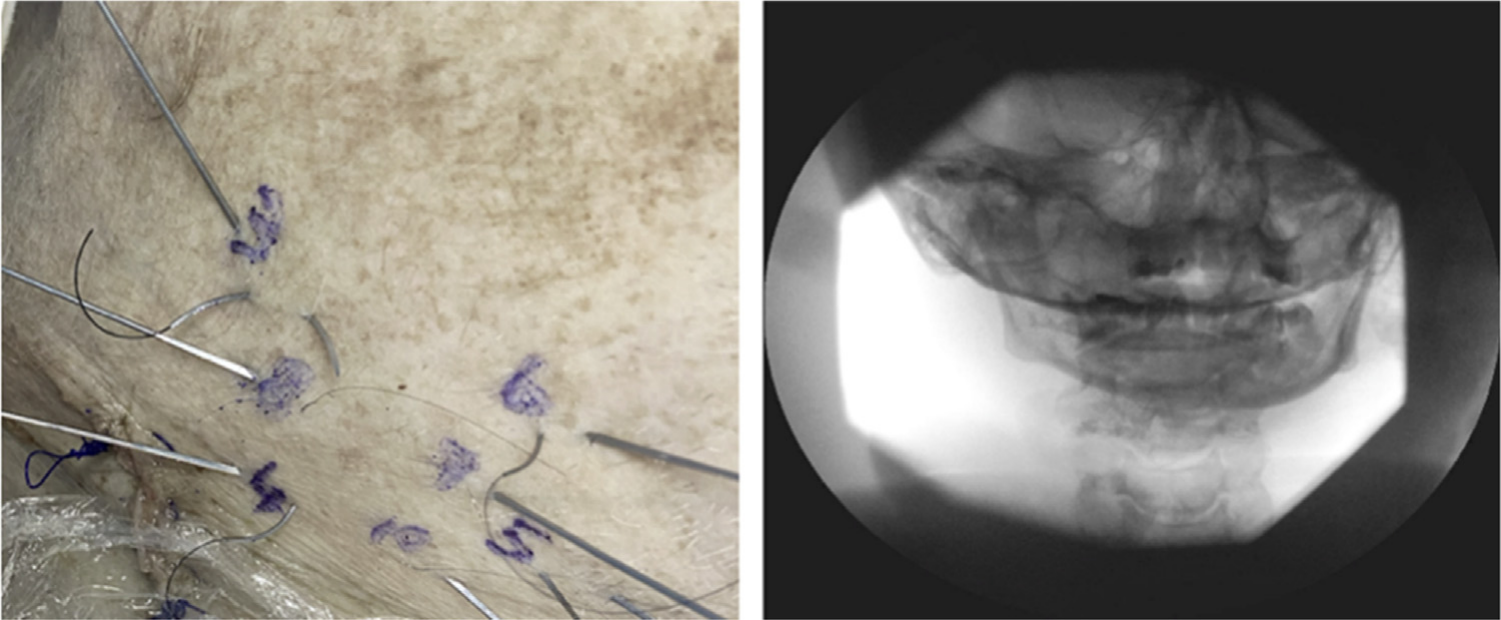
Mandible images/radiograph for needle identification.

**Figure 5:**
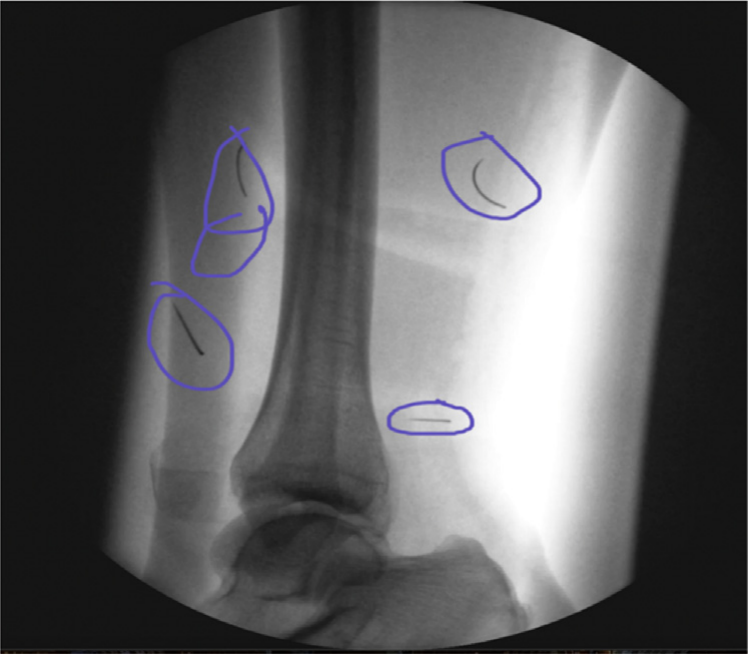
Needle location on radiograph of distal tibia.

The anatomical sites examined included the tibia (Figure 1), distal radius (Figure 2), 2nd intercostal space (Figure 3), and mandible (Figure 4). All needles were removed and disposed of following radiographic acquisition, in compliance with institutional biohazard safety protocols Figure 5.

To assess radiographic detection accuracy, a two-phase evaluation process was used. First, a pilot study was conducted using PowerPoint slides displaying radiographs without reference needles. These were distributed to a convenience sample of consultant surgeons, surgical registrars, radiology trainees, senior house officers, and operating theatre nursing staff. Participants were blinded to the number and caliber of needles present. They were instructed to examine each image and annotate locations where they believed a needle was present using a red circle. The task prompt was: “Please inspect this X-ray and drag a red circle to highlight each point where you see a suture needle.” This phase helped validate image quality and feasibility of the detection task.

Upon completion of the pilot, the main study was implemented using interactive software (www.mindstamp.io © 2022 AECH CO All Rights Reserved). This platform allowed for real-time user interaction and automated data capture. Each participant reviewed four radiographs (one per anatomical site), with each image containing seven suture needles—one of each caliber (3–0 to 10–0, excluding 7–0). Participants were instructed to click directly on the image where they suspected a needle was located. The software logged these click locations and compiled a visual overlay for each image, which was subsequently used to determine accuracy.

A total of 53 participants completed the study, each producing four sets of annotated images. Responses were considered correct if the marked location was within a defined radius of the true needle position, as confirmed by the original placement and imaging coordinates. The results of the pilot participants were pooled with the main study data for final analysis.

Although embalming processes may affect soft tissue characteristics for modalities such as ultrasound, CT, or MRI, existing literature supports the preservation of radiographic integrity in formalin-fixed cadavers.^13^ Therefore, the use of a cadaveric model was deemed valid for simulating intraoperative X-ray imaging conditions. All procedures were performed in accordance with ethical guidelines for the use of human anatomical material in medical research.

## Results

A total of 53 participants completed full responses and therefore were eligible for inclusion in this study. Just under half of the participants were female (41.5%). Responses were considered corrected if participants marked the estimated needle location either accurately on or encircling the needle. The breakdown of specialty is show in Table 2. As there was 53 participants, it was calculate that there was 212 opportunities for each needle caliber to be marked. As each participant reviewed four images each with seven needles present, there was a total of 1484 scores collected.

**Table 2:** Breakdown of number of participant at each level of training.

Specialty/Seniority of participants	Number of participants
Consultant surgeon	10
Surgical trainee SpR/SHO	27
Other surgical NCHD	5
Nursing staff	1
Non-surgical NCHD	8
Radiology trainee	2

A graphically summary of the results is shown in Table 3. The 4–0 and 6–0 Ethilon needles were marked correctly by 100% of the participants across all anatomical locations. Similarly, accuracy in recognition of 3–0 and 5–0 needles from the 4 radiographs was exhibited by 52 participants (99.87%). However, 8–0 suture needles were identified correctly in only 3.77% of marked answers. For 9–0 Ethilon, 0.47% of participants were considered correct, with no 10–0 needles successfully identified.

**Table 3:** Graphically summary of percentages of needles correctly identified by participants relative to needle caliber.

Detection accuracy was consistent across anatomical sites for larger needles but more variable for smaller calibers. Sites with greater soft tissue mass (e.g., anterior chest and neck) showed slightly lower recognition rates for intermediate sizes (6–0, 5–0), though this was not statistically significant.

### Statistical analysis

Descriptive statistics were used to summarize the detection rates for each needle size. Proportions were calculated as the number of correct identifications out of 212 opportunities for each needle caliber. A chi-squared test was used to assess differences in detection rates across needle calibers. The test revealed a statistically significant difference in detection accuracy between needle sizes (χ² = 1135.62, df = 6, *p* < 0.001), confirming that finer needles were significantly less likely to be identified.

Post hoc pairwise comparisons using the Bonferroni correction demonstrated that detection rates for 8–0, 9–0, and 10–0 needles were significantly lower than those for all other needle sizes (*p* < 0.001 for all comparisons). There were no statistically significant differences in detection rates between 3–0, 4–0, 5–0, and 6–0 needles (*p* > 0.05).

Participant performance was also analyzed by specialty; however, due to small subgroup sizes, no statistically significant differences in detection accuracy were observed between surgeons, radiologists, and other healthcare professionals (*p* = 0.14).

These findings indicate that while intraoperative radiography is highly effective in detecting needles of 3–0 to 6–0 caliber, its utility diminishes sharply with smaller, microsurgical needle sizes, reinforcing the need for reconsideration of standard imaging protocols in such contexts.

## Discussion

This study provides important new evidence on the limitations of intraoperative radiography for the detection of retained microsurgical suture needles, particularly those of fine caliber. Retained surgical items (RSIs), although infrequent, are a recognized source of postoperative complications and legal liability, with incidence rates ranging from one in 1000 to one in 19,000 surgical procedures.^7^ Despite the implementation of preventative measures, such as surgical counts and intraoperative imaging protocols, RSIs continue to occur, highlighting the need for continued evaluation and improvement of detection strategies.^1^^,^^2^

Our cadaveric investigation specifically examined the radiographic detectability of microsurgical needles ranging from 3–0 to 10–0 caliber. Detection rates were excellent for larger calibers (3–0 to 6–0), consistent with previously reported data.^2^ However, a dramatic decline in accuracy was observed for 8–0, 9–0, and 10–0 needles, with fewer than 4% of participants identifying the 8–0 needle, only 0.47% identifying the 9–0, and no detection of the 10–0 caliber needle. These findings mirror those of Macilquham et al., who found that while 17 mm needles were reliably detected, only 13% of 13 mm needles were identifiable on radiographs.^2^ The results support the hypothesis that needle size is a critical determinant of radiographic visibility and diagnostic efficacy.

The limitations of intraoperative X-ray imaging become particularly relevant in microsurgical procedures, where 8–0 to 10–0 needles are frequently used for microvascular anastomosis and nerve repair. A clinical survey by McMahon et al. found that 92.5% of reconstructive surgeons had experienced microsurgical needle loss, yet only 15.8% attempted radiographic localization—and none succeeded.^9^ These data underscore the disconnection between established protocols and real-world diagnostic yield. Despite adherence to imaging recommendations, the clinical benefit appears minimal in this context, leading to delays, increased anesthetic time, and heightened procedural complexity.

The significance of these delays is not trivial. Anecdotal data from senior authors (SP, BOS) report operative delays exceeding 60 min following needle loss, largely due to the logistics of acquiring intraoperative radiographs. Such delays are clinically relevant, as prolonged operative time is an independent predictor of microvascular flap failure, as well as increased postoperative complications. Walter et al. reported a false-negative rate of 44% for intraoperative radiographs in detecting retained surgical materials, with sensitivity decreasing further for smaller objects.^14^

In settings where imaging is available, the decision to proceed with intraoperative radiographs should be balanced against the likelihood of needle detection. The clinical decision-making process is further complicated by open disclosure policies such as that of the Health Service Executive (HSE) in Ireland, which mandates that patients be informed of any retained material regardless of harm.^5^ In the absence of robust data showing harm from retained microsurgical needles, this creates a difficult position for surgeons who must justify imaging procedures that are unlikely to yield actionable results.

To our knowledge, this is the first study to use a formalin-embalmed human cadaveric model to evaluate the radiographic detectability of microsurgical needles. This methodological choice enhances anatomical fidelity and better replicates clinical operating conditions compared to previous studies using synthetic or animal tissue.^2^^,^^9^ Importantly, embalming has minimal impact on radiograph quality, although it may alter results for other modalities such as MRI and CT.^13^ These limitations were mitigated through standardized imaging parameters, consistent radiographer use, and minimized object-to-detector distance to improve resolution.

Despite best efforts to simulate intraoperative settings, limitations remain. Participants were instructed to identify needle positions using a point-click interface or by dragging circles over radiographs—methods which, although standardized, introduce subjectivity and observer variability. Future studies may benefit from computer-aided detection tools or radiopaque-enhanced needles.^3^ Additionally, the generalizability of these findings to live operative conditions is limited by the absence of dynamic variables such as tissue perfusion, bleeding, and patient movement.

It is also important to note that this study did not evaluate patient outcomes associated with unretrieved needles. However, a literature review identified no reported cases in which a return to theatre was required solely for retrieval of 9–0 or 10–0 needles.^1^^,^^15^ While case reports exist of needles being detected on MRI or CT postoperatively,^10^^,^^11^ these are isolated and do not support widespread use of advanced imaging in every case of suspected needle loss. Furthermore, computed tomography has been shown to offer improved sensitivity over standard radiography, but its use must be balanced against radiation exposure, cost, and availability^3^^,^^16^ (Table 4).

**Table 4:** Comparison of the advantages and disadvantages of imaging modalities for the detection of lost microsurgical needles.

Modality	Advantages	Disadvantages
Plain radiography (X-ray/C-arm)	• Widely available and cost-effective. • Quick access • Effective at visualizing larger radiopaque needles • C-arm fluoroscopy offers real-time imaging	• Lower sensitivity, especially for smaller needles, a negative x-ray does not reliably rule out retained needle. • 2D images can be obscured by overlapping bone or other structures, making precise 3D localization difficult. • Ionizing radiation exposure for patient and staff.
Computed tomography (CT)	• Considered more accurate for foreign body detection and 3D localization. • Provides detailed, multiplanar images and better tissue density differentiation than x-ray. • Can identify associated complications (e.g., abscesses, hematomas).	• Higher radiation dose compared to x-ray. • Requires patient transport out of the sterile field for conventional scans • Image quality can be diminished by patient movement and metallic artifacts. • High cost and ergonomic challenges for intraoperative CT
Ultrasonography (US)	• Provides real-time, dynamic imaging at the point of care. • No ionizing radiation exposure for patient or staff. • Widely available and cost-effective.	• Effectiveness is highly dependent on the operator • Limited depth penetration, and visualization is hindered by bone or air (e.g., bowel gas). • Challenge identifying very small objects or deep locations.
Magnetic resonance imaging (MRI)	• Better soft tissue contrast and detailed anatomical visualization. • Does not use ionizing radiation.	• Generally contraindicated for metal foreign bodies • High cost, limited intraoperative availability, and long scan times. • Ergonomic and practical barrier for use in operating theatre, therefore would require transfer of patient to scanner • Real- time identification hindered by above

The lack of standardized reporting systems for microsurgical needle loss remains a significant barrier to improving clinical practice.^3^^,^^4^^,^^9^ As detection via standard radiography proves increasingly unreliable for small-caliber needles, there is a compelling need to consider alternative imaging strategies or device modifications, such as embedding radiopaque markers or magnetic alloys.^3^ AI-based image enhancement tools may offer future solutions, and ongoing evaluation of intraoperative CT, ultrasound, and novel technologies should be prioritized in research agendas. Additionally, radiofrequency identification-tagged sponges and barcoding systems have been found to reduce retained surgical sponges, these technologies are not applicable to surgical needles due to size limitations. A dual-purpose fluorescent coating for standard needles has been developed to enable localization using ultraviolet light in open procedures and near-infrared fluorescence during minimally invasive surgery. Ward et al. demonstrated improved needle localization accuracy from 65% to 100% in an animal model. However, this technology requires activation by specific light sources and remains limited when needles are obscured by tissue, necessitating continued manipulation for visualization. Further research is needed to evaluate safety and cost-effectiveness of this technology before implementing it into the operating theatre.^3^^,^^9^^,^^15^

Collectively, these findings suggest that while current X-ray-based protocols may be adequate for standard suture needles (≥6–0), they are largely ineffective for finer microsurgical instruments. Adopting a more selective, risk-based imaging protocol may help optimize resource allocation, reduce intraoperative delays, and align practice more closely with evidence-based medicine.

## Conclusion

This study demonstrates that while intraoperative radiography is highly reliable for detecting standard-caliber suture needles (3–0 to 6–0), its diagnostic performance declines sharply for finer microsurgical needles, particularly those of 8–0 caliber and smaller. Detection rates for 8–0, 9–0, and 10–0 needles were exceedingly low, with no 10–0 needles identified by any participant. These findings have important implications for surgical protocols: routine use of portable X-ray imaging in response to microsurgical needle loss may not be justified, given the minimal likelihood of successful detection and the associated costs, delays, and radiation exposure.

Institutions should consider revising existing RSI protocols to reflect the limited utility of X-ray imaging for very fine needles, particularly when clinical risk is low and no adverse events have been reported in the literature. A more selective, evidence-based approach—potentially incorporating alternative imaging modalities or enhanced detection technologies—may better support surgical decision-making. Ultimately, these findings reinforce the need for pragmatic, risk-stratified management of microsurgical needle loss, balancing patient safety, resource use, and operative efficiency.

## Ethical statement

Ethical approval for this study was obtained from both the Mater Misericordiae University Hospital (MMUH) and University College Dublin (UCD).

## Declaration of competing interest

Not declared.
